# Safety of Human USH1C Transgene Expression Following Subretinal Injection in Wild-Type Pigs

**DOI:** 10.1167/iovs.66.1.48

**Published:** 2025-01-21

**Authors:** Peter Kiraly, Joshua Klein, Immanuel P. Seitz, Felix F. Reichel, Tobias Peters, Taras Ardan, Jana Juhasova, Stefan Juhás, Zdenka Ellederova, Yaroslav Nemesh, Ruslan Nyshchuk, Nikolai Klymiuk, Kerstin Nagel-Wolfrum, Ashley R. Winslow, Uwe Wolfrum, Jan Motlik, M. Dominik Fischer

**Affiliations:** 1 Oxford Eye Hospital, Oxford University Hospitals NHS Foundation Trust, Oxford, United Kingdom; 2 Nuffield Laboratory of Ophthalmology, University of Oxford, Oxford, United Kingdom; 3 Institute of Molecular Physiology, Molecular Cell Biology, Johannes Gutenberg University, Mainz, Germany; 4 University Eye Hospital Tübingen, Centre for Ophthalmology, University of Tübingen, Tübingen, Germany; 5 STZeyetrial GmbH, Tübingen, Germany; 6 Institute of Animal Physiology and Genetics, Czech Academy of Science, Libechov, Czech Republic; 7 Department of Cell Biology, Faculty of Science, Charles University, Prague, Czech Republic; 8 Large Animal Models in Cardiovascular Research, Internal Medical Department I, TU Munich, Munich, Germany; 9 Institute of Developmental Biology and Neurobiology (iDN), Johannes Gutenberg University, Mainz, Germany; 10 Odylia Therapeutics, Atlanta, Georgia, United States

**Keywords:** usher syndrome, USH1C, gene therapy, wild type pigs

## Abstract

**Purpose:**

This study aimed to evaluate early-phase safety of subretinal application of AAVanc80.CAG.USH1Ca1 (OT_USH_101) in wild-type (WT) pigs, examining the effects of a vehicle control, low dose, and high dose.

**Methods:**

Twelve WT pigs (24 eyes) were divided into three groups: four pigs each received bilateral subretinal injections of either vehicle, low dose (3.3 × 10^10^ vector genomes [vg] per eye), or high dose (1.0 × 10^11^ vg per eye). Total retinal thickness (TRT) was evaluated using optical coherence tomography and retinal function was assessed with full-field electroretinography (ff-ERG) at baseline and two months post-surgery. After necropsy, retinal changes were examined through histopathology, and human *USH1C_a1*/harmonin expression was assessed by quantitative PCR (qPCR) and Western blotting.

**Results:**

OT_USH_101 led to high *USH1C_a1* expression in WT pig retinas without significant TRT changes two months after subretinal injection. The qPCR revealed expression of the human *USH1C_a1* transgene delivered by the adeno-associated virus vector. TRT changes were minimal across groups: vehicle (256 ± 21 to 243 ± 18 µm; *P* = 0.108), low dose (251 ± 32 to 258 ± 30 µm; *P* = 0.076), and high dose (242 ± 24 to 259 ± 28 µm; *P* = 0.590). The ff-ERG showed no significant changes in rod or cone responses. Histopathology indicated no severe retinal adverse effects in the vehicle and low dose groups.

**Conclusions:**

Early-phase clinical imaging, electrophysiology, and histopathological assessments indicated that subretinal administration of OT_USH_101 was well tolerated in the low-dose treatment arm. OT_USH_101 treatment resulted in high expression of human *USH1C_a1*. Although histopathological changes were not severe, more frequent changes were observed in the high-dose group.

Usher syndrome (USH) is a ciliopathy with an autosomal recessive inheritance pattern, characterized by sensorineural hearing impairment, retinitis pigmentosa (RP), and in some cases, vestibular dysfunction.^1^ It is a primary cause of concurrent deafness and blindness, accounting for more than 50% of such cases.^2^^,^^3^ The prevalence of USH ranges from 3.2 to 6.2 per 100,000 individuals,^1^ and it represents 18% of all RP^3^ and 5% of all congenital hearing loss cases.^4^ USH is divided into four clinical types (USH1, USH2, USH3, USH4), based on the visual impairment and age of RP onset, the severity and onset of hearing loss, and the presence of vestibular dysfunction.^5^^–^^7^ USH is genetically heterogeneous with 11 causative genes confirmed and two suspected.^8^ Mutations in *USH1C* are responsible for 6% to 7% of USH1 cases,^1^ which make up one third of all USH patients and represent its most severe clinical subtype.^9^ The *USH1C* gene encodes various isoforms of the harmonin protein, which organize protein networks within the USH interactome.^10^^,^^11^ Hearing aids and cochlear implants are the main treatments for hearing loss in USH, but there is currently no approved treatment for the USH-related vision loss.^12^ Since the approval of voretigene neparvovec for biallelic RPE65-mediated inherited retinal dystrophies, there has been significant advancement in ocular gene therapy, evidenced by numerous ongoing pre-clinical and clinical trials.^13^^,^^14^ In USH, several therapeutic approaches are being investigated including DNA interventions (gene augmentation, gene editing), RNA interventions (antisense oligonucleotides [ASOs], translational readthrough) and cell therapies.^15^ Currently, there are no human interventional trials specifically for patients with *USH1C* mutations, except for an antioxidant drug trial by Nacuity Pharmaceuticals enrolling USH patients regardless of mutation type.^16^ In 2007, Lentz et al. developed a knock-in mouse model with the human 216G > A mutation (a founder mutation in North America), that exhibits severe hearing loss, balance problems, and mild vision loss.^17^ Systemic treatment with an ASO targeting the 216G > A mutation rescued hearing and vestibular function in the mouse model.^18^ In the same mouse model, Pan et al.^19^ observed that following the delivery of wild-type *USH1C* into the inner ear using an adeno-associated viral vector (AAV), there was gene and protein expression, resulting in the recovery of auditory and vestibular function. In 2022, Grotz et al.^20^ developed a transgenic humanized USH1C^R31*^ pig model by inserting a human *USH1C* gene mutation, effectively replicating key USH1 symptoms such as hearing loss, balance issues, and vision problems. This large animal model provided valuable insights into USH1C disease development and supported the establishment of a breeding herd for gene therapy trials.^20^

Determining dose-limiting toxicity is an important step in the development of gene therapies for inherited retinal dystrophies. This study aimed to assess the safety and feasibility of subretinal application of AAVanc80.CAG.USH1C_a1 (OT_USH_101) in wild-type (WT) pigs, evaluating the effects of a vehicle control, low dose, and high dose, to identify the optimal dose for future treatment in the humanized USH1C^R31*^ pig model.^20^

## Methods

**Figure 1.**
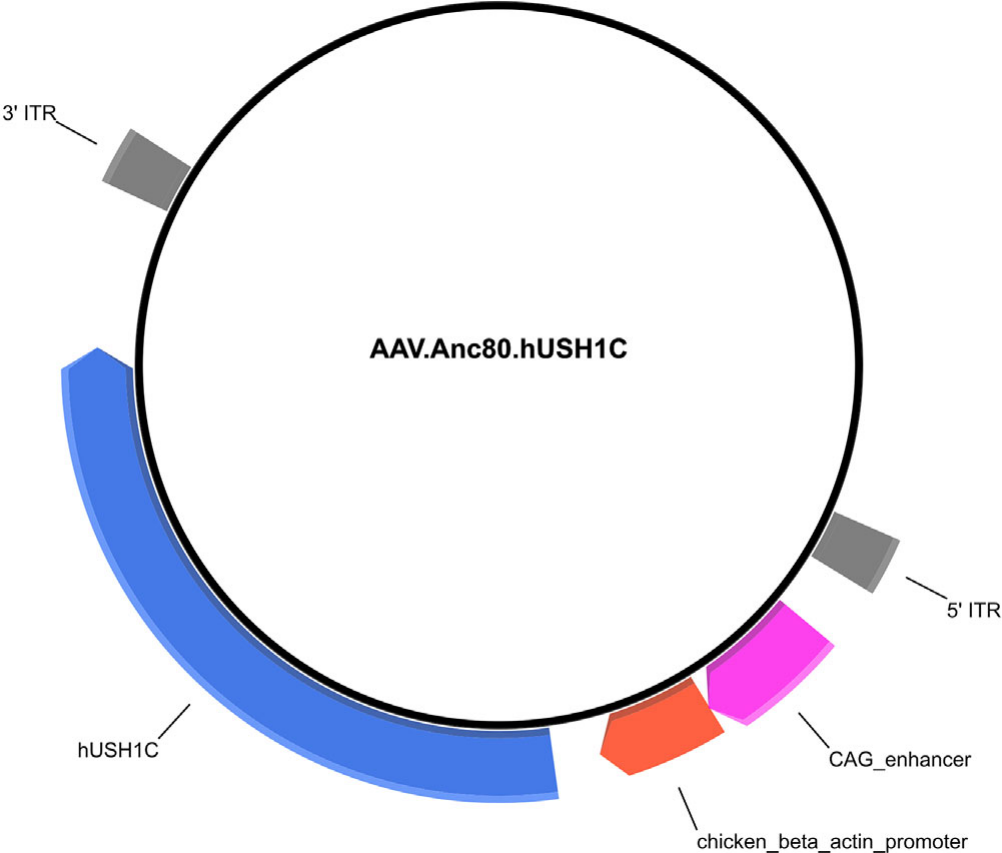
Schematic representation of the design of the OT_USH_101 adeno-associated viral vector.

The studies adhered to the ARVO guidelines for animal use in ophthalmic and vision research and conformed to the Czech Republic's Animal Protection Law and received approval from the Ethics Committee of the Czech Academy of Sciences, Prague, Czech Republic (Approval No. IAPG CAS CZ/790/2019, dated June 20, 2019). Surgeries and experiments were performed at the PIGMOD Center, Liběchov, Czech Republic. Twelve male WT pigs (24 eyes) of the Liběchov minipig strain^21^ were allocated into three groups: each group of four pigs received bilateral subretinal injections of 50 µl volume per eye containing either vehicle (1 × PBS + 35 mM NaCl + 0.001% PF-68), OT_USH_101 low dose (3.3 × 10^10^ vg per eye), or OT_USH_101 high dose (1.0 × 10^11^ vg per eye). Endotoxin-free OT_USH_101 was provided by Odylia Therapeutics and produced at the Harvard Vector Core Facility. Schematic representation of AAV design is presented in Figure 1. On the day of the procedures, the appropriate number of vials was taken out of storage (−80°C) and allowed to thaw at room temperature. Aliquots were pooled and a working solution for each treatment arm was prepared under a laminar flow hood using sterile technique. Final concentrations were achieved by diluting stock solutions with vehicle (1 × PBS +35 mM NaCl + 0.001% PF-68) to allow delivery of 0 vg per eye (vehicle), 3.3 × 10^10^ vg per eye (low dose), or 1.0 × 10^11^ vg per eye (high dose) in a volume of 50 µL injected into the subretinal space.

### Ocular Surgery

WT pigs were 3 ± 1 months old and weighed approximately 30 kg at the time of surgery. General anesthesia was induced by intramuscular injection of azaperone (2 mg/kg), atropine (8 µg/kg), and ketamine (20 mg/kg). Atracurium (0.67 mg/kg) was given prior to intubation and anesthesia was maintained with continuous propofol perfusion (1% at 20 ml/h). Before surgery, the periorbital regions were thoroughly cleaned with povidone iodine, and sterile surgical drapes were applied. A lid speculum was then placed, and three sterile small-gauge trocars (23G) were inserted through the pars plana. After performing a vitrectomy, a localized retinal detachment was induced by subretinal injection using a small gauge cannula, and approximately 0.05 mL of the solution was applied to the subretinal space targeting the visual streak. Injectable AAV solution or vehicle was delivered into the subretinal space by a handheld 41G extendable cannula (DORC 1270.EXT). All surgeries were performed by a single experienced surgeon (MDF) to ensure the best possible consistency across groups. After the subretinal injection, the trocars were removed, and a 0.05 mL solution containing dexamethasone (1 mg) was applied under the conjunctiva, and 0.3% ofloxacin ointment was applied to the surface of the eye before the animal's recovery. For postoperative care, animals received 0.5% moxifloxacin and 1% prednisolone eye drops three times daily for a week. Systemic immunosuppression included prednisone at 1 mg/kg (intramuscular) from day 2 until day 5, followed by 0.5 mg/kg for the next seven days, and then 0.25 mg/kg for another seven days.

### Optical coherence tomography

**Figure 2.**
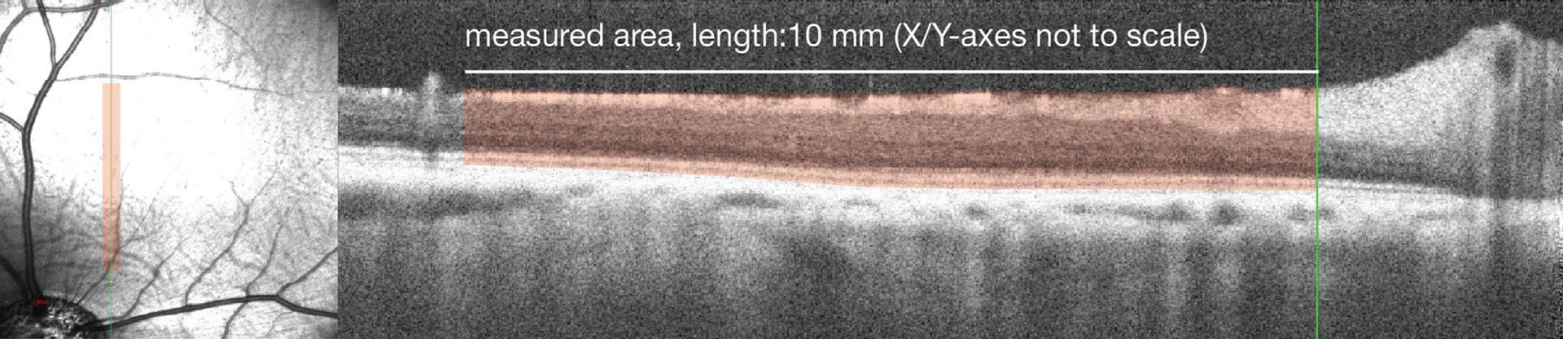
Optical section through the visual streak of the treated WT pig retina. On the left: Scanning Laser Ophthalmoscopy (SLO) image showing the placement of the B-scan. On the right: Optical coherence tomography (OCT) B-scan with the marked area for analysis.

Optical coherence tomography (OCT) scans were obtained with the animals under general anesthesia just before surgery and two months after the surgery. Retinal thickness was determined as the span between the inner boundary of the internal limiting membrane and the outer boundary of the retinal pigment epithelium (RPE). The mean total retinal thickness (TRT) was quantified using spatially reproducible B-scans, aligned tangentially to the temporal edge of the optic nerve head, consistently crossing the visual streak, and encompassing a significant portion of the treated area. After manually measuring the marked area and the length of the x-axis as shown in Figure 2, TRT was calculated using the formula provided below.

$$\begin{array}{c} \mathrm{mean}\;\mathrm{TRT}\;\left[µm\right]=\frac{\mathrm{marked}\;\mathrm{area}\;\left[µm^{2}\right]}{\mathrm{area}\;\mathrm{length}\;\mathrm{along}\;x-\mathrm{axis}\;\left[µm\right]} \end{array}$$

### Full-Field Electroretinography (ff-ERG) Recordings

Ff-ERG (Roland Consult, Brandenburg an der Havel, Germany) measurements were obtained using a corneal Kooijman electrode, a reference electrode at the temple, and ground electrode on the top of the head between the ears. After pupil dilation, measurements were obtained with the animals under general and topical anesthesia, just prior to surgery and two months after surgery, in the right eye only. To assess cone function, we analyzed the ISCEV standard light-adapted 3.0 ERG and the light-adapted 3.0 flicker ERG responses. To assess the rod system and mixed responses, we applied a white flash intensity series following 30 minutes of dark adaptation (0.01, 0.03, 0.1, 0.3, 1, and 3 cd/m^2^).

### Histopathology

Initially, eyes were fixed in Davidson's fixative for 24 hours, followed by post-fixation in buffered 4% formalin for storage. The samples were then dehydrated through an ethanol series and embedded in paraffin using an automatic tissue infiltrator (TP 1020; Leica Wetzlar, Germany). Sectioning was performed with a microtome (RM2255, Leica Wetzlar, Germany), with subsequent dewaxing and hematoxylin and eosin staining. A semiquantitative preclinical and clinical histopathology was assessed in the sections in a masked fashion, scoring (0 = no; 1 = minimal; 2 = slight; 3 = moderate; 4 = severe) the following parameters: retinal atrophy, retinal detachment, retinal vacuolation, retinal (peri)vasculitis, choroidal (peri)vasculitis, inflammatory cells (choroid), inflammatory cells (retina), hemorrhages (choroid), hemorrhages (retina), irregular pigmentation.

### Tissue Analysis of *USH1C*/Harmonin Expression

The expression of *USH1C*/harmonin was evaluated in retinal areas where a bleb had previously been raised, as well as in corresponding untreated retinal areas. The samples were prepared by dissecting retinal tissue using an 8 mm punch and then immediately snap-freezing them in liquid nitrogen. The expression was quantitatively assessed using two main methods. First, quantitative real-time PCR was performed to specifically measure the transcript expression levels of the human *USH1C* in porcine retinas (Quantstudio 1 Real-Time-PCR-System; Thermo Fisher Scientific, Waltham, MA, USA). The 8 mm retinal punch samples were used as a source for mRNA isolation, followed by reverse transcriptase reactions and quantitative PCR (qPCR) (40 cycles). Interspecies differences in *USH1C* (ortholog) sequence in exons 9-11 were used to select a primer combination capable of discriminating between human *USH1C* (Fwd.: ATCCAGAAGCCTGGCATCTTTATCA, Rev.: GCGGCTACTCTTCAGCACATTTACA) and porcine *Ush1c* (Fwd.: GTCCAGAAACCCGGCATATTCATCA, Rev.: ACGGCTACTTTTCAGCACGTTCACG).

Second, Western blot analysis was conducted, enabling us to compare protein expression levels between the treated and untreated regions of porcine retinas. For this, frozen retina samples, stored at −80°C, were processed as previously described.^22^ In brief, retinal tissues were lysed in modified RIPA buffer (50 mM Tris–HCl, 150 mM NaCl, 0.1% SDS, 2 mM EDTA, 1% NP-40, 0.5% sodium-deoxycholate, 1 mM sodium vanadate, 30 mM sodium-pyrophosphate, pH 7.4). Protein lysates were separated by SDS-PAGE gel electrophoresis, followed by semi-dry Western blotting. Western blots were analyzed with the Odyssey infra-red imaging system (LI-COR Biosciences, Lincoln, NE, USA). For Western blots, previously validated rabbit polyclonal H3 antibodies targeting the N-terminal of harmonin (1:1000)^23^ and rabbit polyclonal antibodies against glial fibrillary acid protein (GFAP; no. ZO334; DAKO by Agilent, Santa Clara, CA, USA; 1:10,000), were used in combination with moose monoclonal antibodies against actin (no. Sct MA5-11869; Invitrogen, Carlsbad, CA, USA; 1:2000) as loading control.

### Immunohistochemistry

Punches of porcine retinas were fixed in melting isopentane for cryosectioning as previously described.^24^ In brief, retinal punches were sectioned in a MICROM HM 560 Cryo-Star cryostat (Thermo Fisher Scientific) and after blocking incubated over-night at 4°C with rabbit polyclonal antibodies against GFAP (no. ZO334; 1:1,000). After washing cryosections were incubated with secondary antibodies and counterstained for nuclear DNA with DAPI (4’,6-diamidino-2-phenylindole) (1 mg/mL) (Sigma-Aldrich, St Louis, MO, USA) for one hour at room temperature. Sections mounted in Mowiol (Roth, Karlsruhe, Germany) were analyzed on a Leica DM6000B microscope (Leica, Bensheim, Germany); documented images were processed with LAS-AF Leica deconvolution and ImageJ/Fiji or Adobe Photoshop CS (Adobe Systems, San Jose, CA, USA) software.

## Results

**Figure 3.**
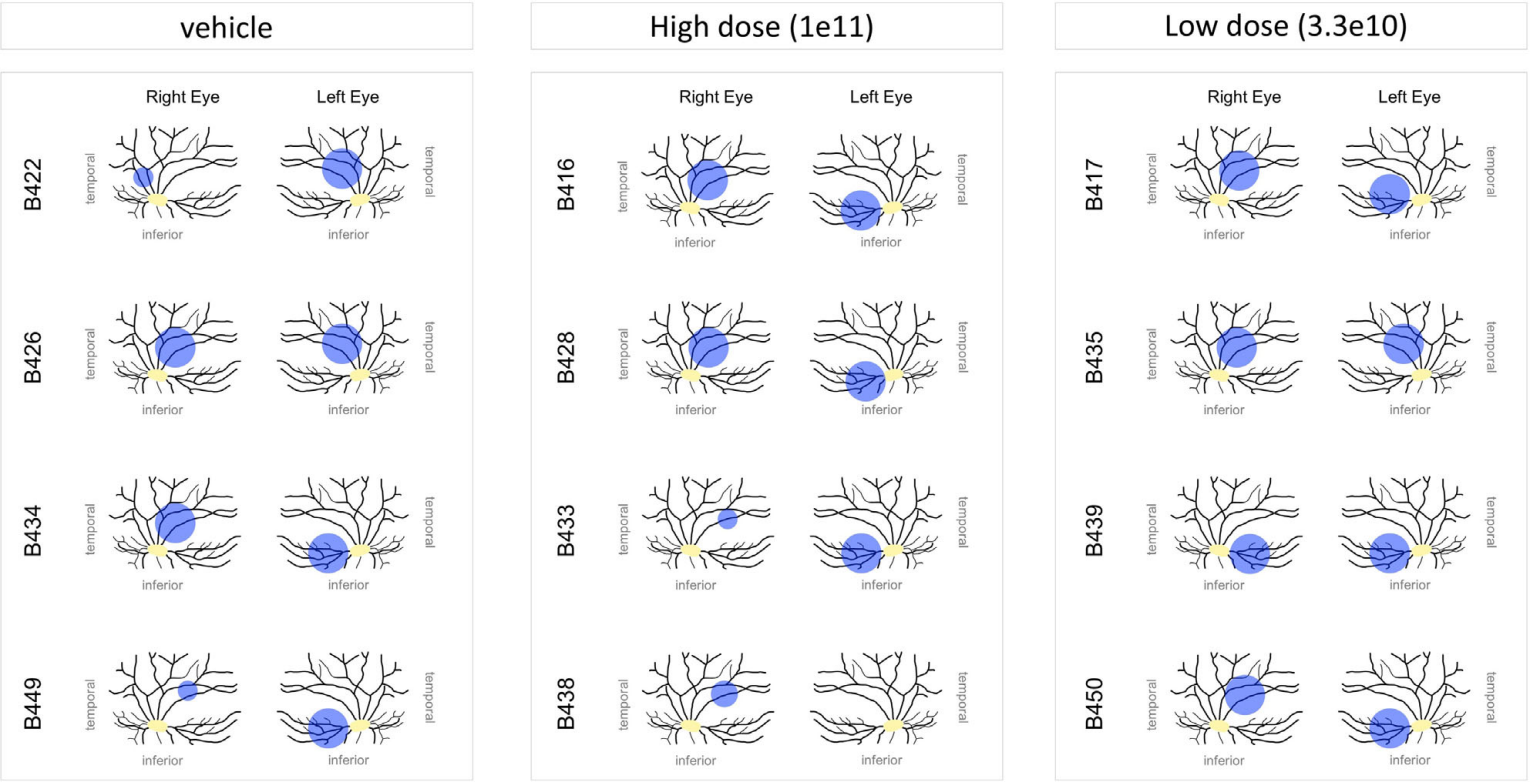
Anatomical location of the raised subretinal bleb (*blue*) related to the vascular system (*black lines*) and the optic nerve head (*yellow*) after subretinal administration of the vector targeting the visual streak of all 24 WT pig eyes.

**Figure 4.**
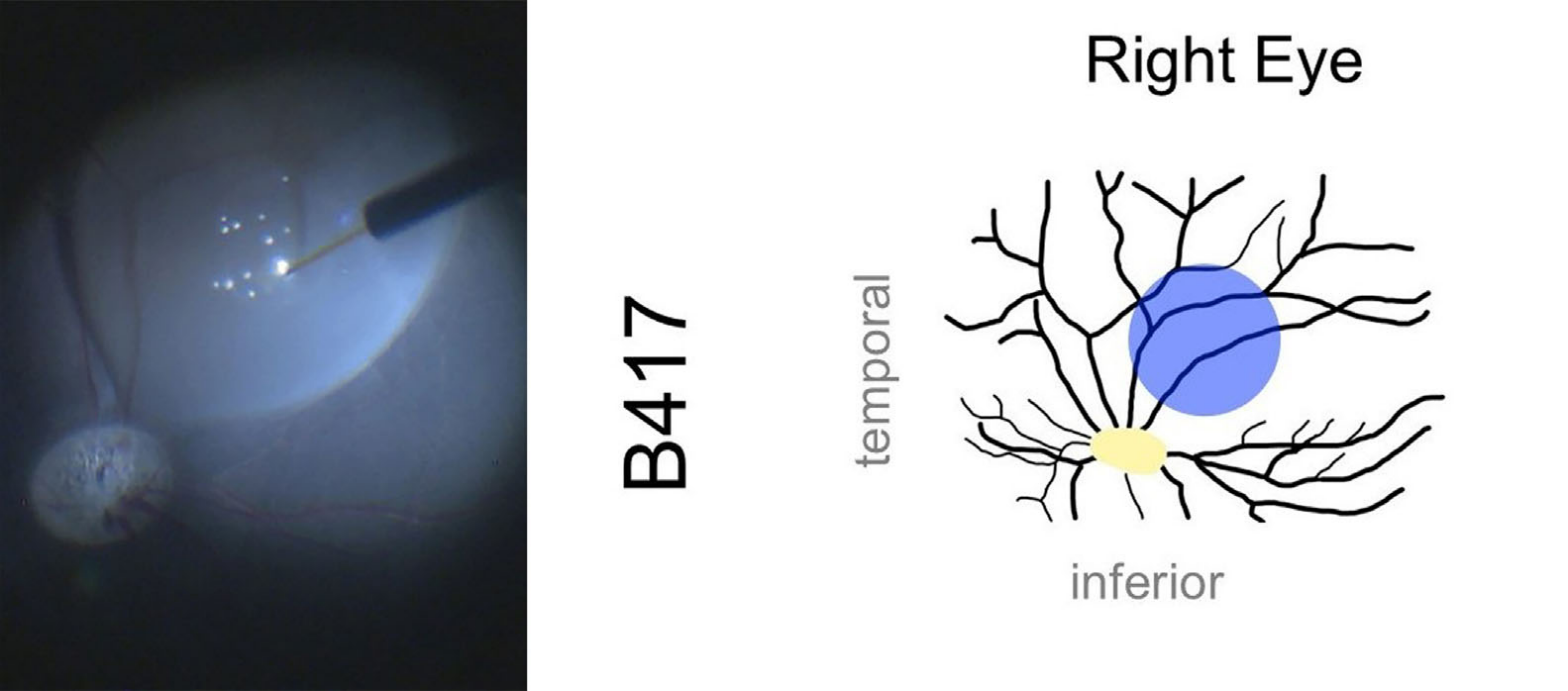
Intraoperative image of a raised subretinal bleb by subretinal injection of the vector and the anatomical location of the bleb (*blue*) within the visual streak of the right eye from animal B417.

All 12 WT pigs were treated as planned, with four animals per group receiving bilateral single subretinal injections. At least five eyes per group were treated with the intended dose/volume without any complications: six out of eight in the vehicle group, five out of eight in the high dose group, and all eight out of eight in the low dose group. Figure 3 illustrates the anatomical location of the raised subretinal bleb in all 24 eyes, whereas Figure 4 presents an example of an intraoperative image showing a raised subretinal bleb.

### OCT Analysis Reveal No Significant Change in TRT After Subretinal Vector Injections

Regarding morphological changes on OCT, none of the cohorts exhibited a statistically significant change in TRT two months after the subretinal injection. In the vehicle group, three WT pigs were used for analysis, whereas in the low- and high-dose groups, four WT pigs were used. One WT pig in the vehicle group was excluded from the analysis because OCT scans were not available two months post-surgery because of technical difficulties with the OCT imaging platform. Specifically, in the vehicle group, the average TRT changed from 256 ± 21 to 243 ± 18 µm after two months (*P* = 0.108); in the low-dose group, it changed from 251 ± 32 to 258 ± 30 µm (*P* = 0.076); and in the high-dose group, it changed from 242 ± 24 to 245 ± 28 µm (*P* = 0.590) (Table 1).

**Table 1.** TRT Changes Before Surgery (Baseline) and Two Months After Surgery

	Baseline (Average ± 2 SD)	Month 2 (Average ± 2 SD)	*P* Value
Vehicle, n = 3	256 ± 21 µm	243 ± 18 µm	0.108
Low dose (3.3 × 10^10^ vg), n = 4	251 ± 32 µm	258 ± 30 µm	0.076
High dose (1.0 × 10^11^ vg), n = 4	242 ± 24 µm	245 ± 28 µm	0.590

n, number; SD, standard deviation; vg, vector genome

p = statistical significance between baseline and two months post-surgery (two-sided paired-sample t-test).

### Full-Field ERG Recordings Reveal No Significant Alterations in Response Derived From Rods and Cones After Subretinal Vector Injections

**Figure 5.**
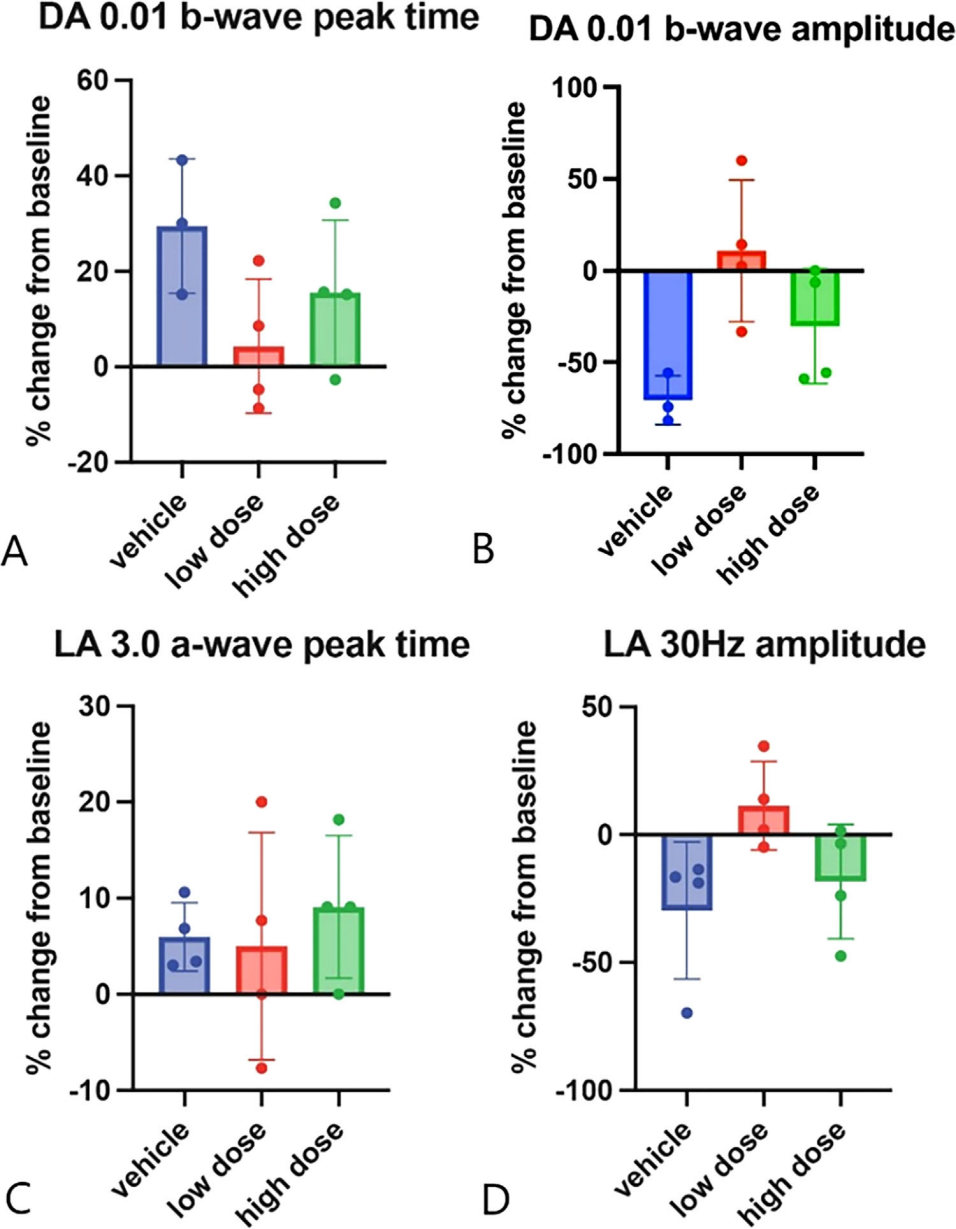
Percentage changes in ERG readings from baseline (before surgery) to two months post-surgery. Top panels (**A, B**) show analysis of rod-dominated responses (dark-adapted 0.01 cd/m^2^) ERG: Percentage changes in b-wave peak time (**A**) and amplitude (**B**) between baseline and two months indicate no global toxicity to rod photoreceptors in either treatment group with the vehicle group performing worst (increase in peak time and loss of amplitude). Bottom panels (**C, D**) display cone-dominated responses: Percentage changes in a-wave peak time (**C**) following a single flash (light-adapted 3.0 cd/m_2_) ERG and percentage changes in amplitude (**D**) in the light-adapted 30 Hz flicker at 3 cd/m^2^ between baseline and two months, showing no global toxicity to the cone photoreceptor population in either treatment group.

When comparing a-wave and b-wave amplitudes and peak times of ff-ERGs across the cohorts in both photopic and scotopic measurements, we observed no significant changes indicative of toxicity in the responses driven by either rods or cones (Fig. 5). For the cone-dominated responses (Figs. 5C, 5D), four WT pigs were analyzed in all groups, whereas to assess rod responses (Figs. 5A, 5B), three WT pigs were analyzed in the vehicle group, and four WT pigs in the low- and high-dose groups. Samples of ERG traces from baseline (before surgery) to the two-month post-surgery follow-up are shown in Supplementary Figure S1.

### Histopathology Showed No Severe Adverse Retinal Changes After Subretinal Vector Injections

**Figure 6.**
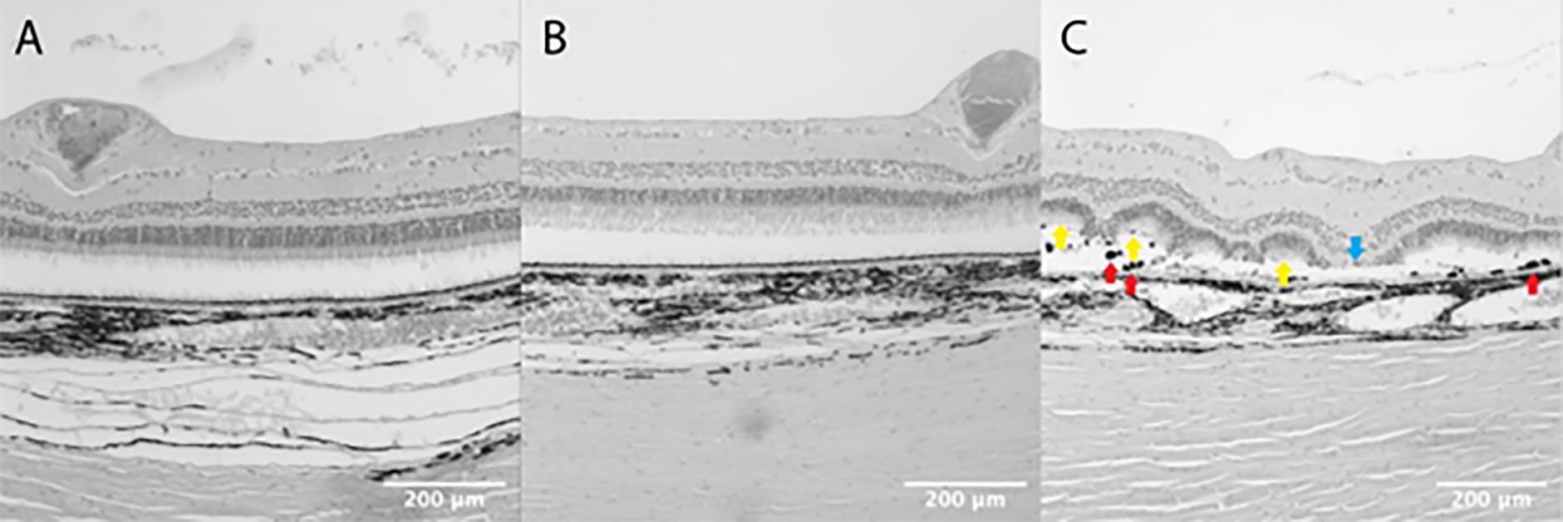
Wide field microscopy images of treated areas of WT pig retinas. No retinal changes suggestive of toxicity or inflammation were observed in the histopathological analysis in a WT pig in the vehicle group (**A,** pig B449) and the low dose group (**B,** pig B417). In the high dose group, pigmentary clumps (*red arrows*), atrophy of the outer retinal layers (*blue arrow*), and rosettes (*yellow arrows*) were seen in pig B416 (**C**).

Histopathology indicated no severe adverse retinal effects in the vehicle and low dose groups, although the high-dose group led to an increased frequency and severity of test-item-related changes. Specifically, there were more inflammatory changes in the outer retinal layers in animals treated with the high dose (Table 2; Fig. 6). In the high-dose group, the right eye of animal B416 showed moderate multifocal inflammatory and atrophic changes after receiving 1.0 × 10^11^ vg in 0.05 mL. The left eye of B433 (high dose) displayed similar, albeit minimal, changes. Among low-dose animals, only one (B439) exhibited minimal adverse changes in the left eye. The vehicle group animals showed no adverse changes on clinical histopathological assessment. All serial sections revealed nearly complete retinal detachment, deemed an artifact from processing, because pre-sacrifice OCT imaging showed attached retinas. Fixation with 4% paraformaldehyde caused artifacts such as retinal and choroidal expansion, splitting, tearing, folding, choroidal pigment clumping, and lens fragmentation and shriveling in all examined animals.

**Table 2.** Changes in Chorioretinal Histopathology in Vehicle, Low-Dose, and High-Dose Groups

Group, Laterality	Animal Number	Retinal Atrophy	Retinal Detachment	Retinal Vacuolation	Retinal (Peri) Vasculitis	Choroidal (Peri) Vasculitis	Inflammatory Cells (Choroid)	Inflammatory Cells (Retina)	Haemorrhages (Choroid/Retina)	Irregular Pigmentation
High dose, L	B428	0	4	0	0	0	0	0	0	0
High dose, R	B416	2	4	1	0	2	2	2	0	3
High dose, L	B433	1	4	0	0	1	0	0	0	1
Low dose, L	B435	0	4	0	0	0	0	0	0	0
Low dose, R	B417	0	4	0	0	0	0	0	0	0
Low dose, L	B439	0	4	1	0	0	0	0	0	1
Vehicle, L	B449	0	4	0	0	0	0	0	0	0
Vehicle, L	B434	0	4	0	0	0	0	0	0	0
Vehicle, R	B426	0	4	0	0	0	0	0	0	0

0 = no; 1 = minimal; 2 = slight; 3 = moderate; 4 = severe changes

### Analysis of the Human USH1C Transgene Expression

**Figure 7.**
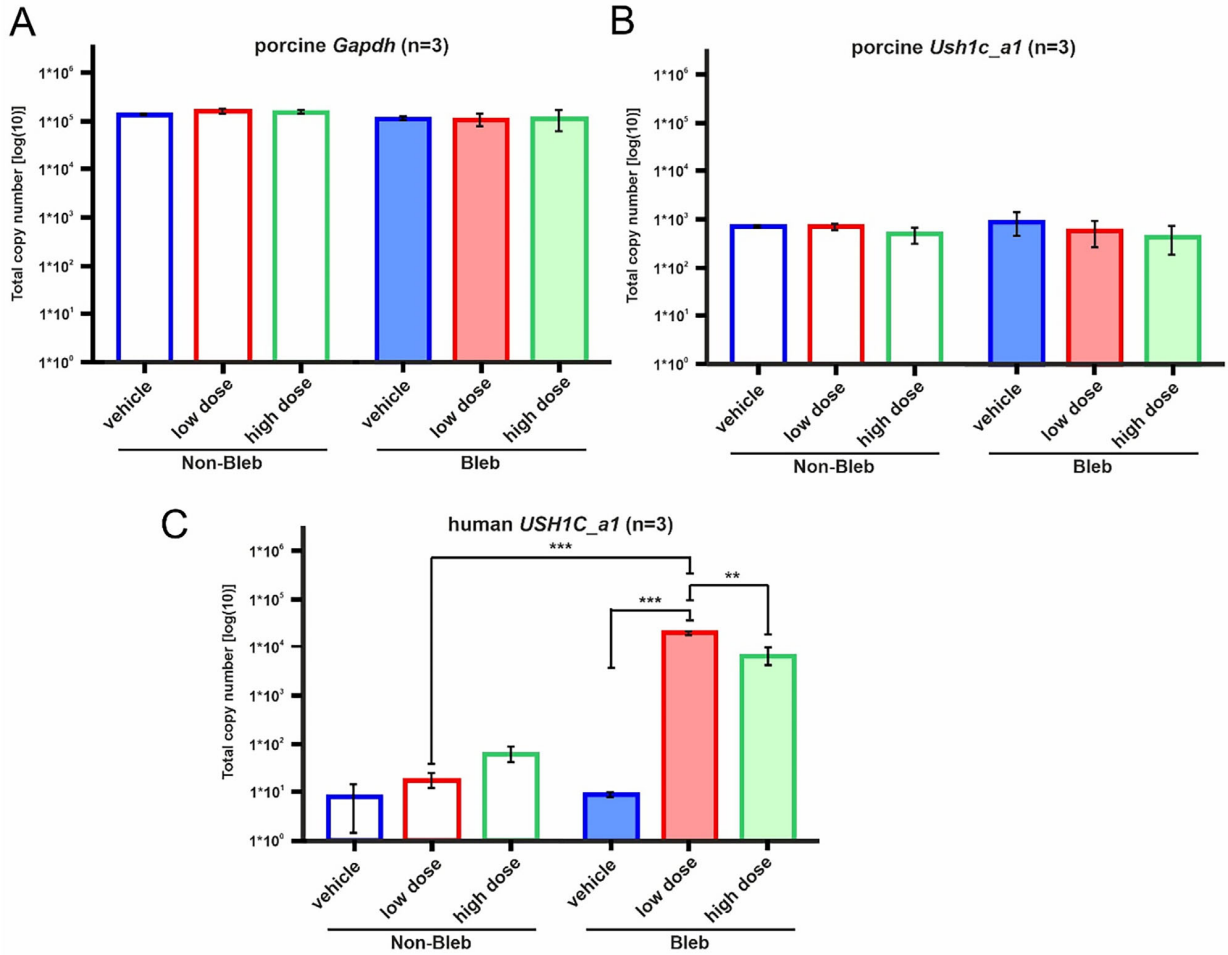
Expression analysis of porcine *Gapdh*, porcine *Ush1c,* and human *USH1C* mRNA in wild type porcine retinas after application of vehicle (*blue*) and vectors at low (*red*) and high (*green*) doses. (**A**) QPCR analysis of mRNA expression of porcine *Gapdh* in the non-treated (non-bleb) compared to treated (bleb) areas of retinas. (**B**) QPCR analysis of mRNA expression of porcine *Ush1c* in the non-treated (non-bleb) compared to treated (bleb) areas of retinas. (**C**) QPCR analysis of mRNA expression of human *USH1C* in the non-treated (non-bleb) compared to treated (bleb) areas of retinas. Significant differences were observed only as increases in transgenic human *USH1C* mRNA expression following low dose treatment (two-way analysis of variance; *** *P* < 0.001; ** *P* = 0.005).

Sampling of treated (bleb) versus untreated (non-bleb) areas was successfully performed in all animals by punch biopsy after identifying treated and non-treated areas based on the surgical notes and confirming them macroscopically in the porcine retina landscape. RNA extraction from half of the 8 mm biopsy punch yielded an average of 56 ng of total RNA per punch. Porcine *Gapdh* was chosen as housekeeping gene and the *Gapdh* expression levels were similar across all groups (Table 3, Fig. 7). The same was the case for the expression levels of porcine *Ush1c* if compared between the treatment arms. Samples from the treated areas following successful subretinal delivery of OT_USH_101 showed expression of human *USH1C_a1* transcripts in WT porcine retina. The expression of human *USH1C_a1* is significantly increased after the low dose injection (Table 3; Fig. 7). However, in the high-dose group expression levels strongly varied among the animals, possibly related to significant reflux of vector suspension noted in two out of three samples from the high-dose group (B433 and B438, see Fig. 3). Interestingly, samples from the treated areas after successful subretinal delivery of OT_USH_101 showed similar levels of porcine *Ush1c* mRNA expression compared to untreated areas, suggesting that the episomally derived human harmonin a1 expression does not impact expression levels of the porcine orthologue.

**Table 3.** Mean Total Copy Numbers Amplified for Porcine *Gapdh*, Porcine *Ush1c*, and Human *USH1C*, Which Entered the Quantifications in Figure 7

Gene	Treatment	Non-Bleb	Bleb
Porcine *Gapdh*	Vehicle	132,084.89	115,243.70
	Low dose	154,007.44	112,335.60
	High dose	146,594.94	116,054.40
Porcine *Ush1c*	Vehicle	719.28	930.11
	Low dose	705.89	592.44
	High dose	495.83	460.67
Human *USH1C*	Vehicle	8.22	9.06
	Low dose	18.44	21,799.61
	High dose	63.28	6,849.00

**Figure 8.**
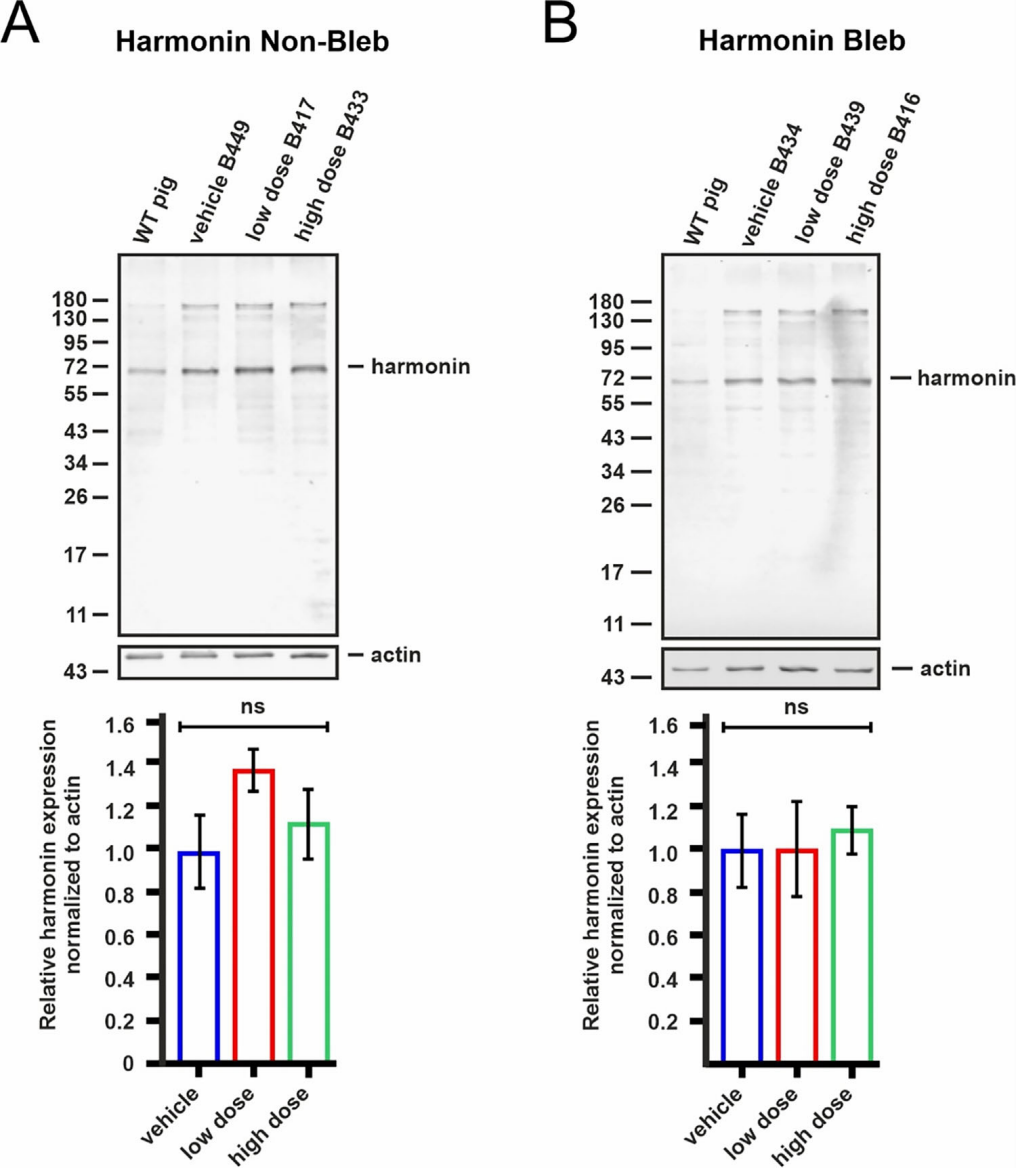
USH1C/harmonin protein expression analysis of porcine retinas after application of vehicle (*blue*) and vectors at low (*red*) and high (*green*) doses. (**A**) Representative anti-harmonin Western blot of protein lysates from the non-treated (non-bleb) areas of porcine retinas; lower bar plot: quantification of anti-harmonin bands relative to the anti-actin loading control bands in three Western blots from three porcine retinas from different animals. (**B**) Representative anti-harmonin Western blot of protein lysates from the treated (bleb) areas of porcine retinas; lower bar plot: quantification of anti-harmonin bands relative to the anti-actin loading control bands in three Western blots from three porcine retinas from different animals. No significant increase in harmonin protein expression was observed in the non-treated (non-bleb) and treated (bleb) areas of retinas treated with low and high vector doses (one-way analysis of variance, ns = not significant).

The other half of each 8 mm retina punch was used for USH1C/harmonin protein expression analysis. Western blot analysis was performed using previously validated rabbit polyclonal H3 antibodies targeting the N-terminal of mouse harmonin.^23^ Because the N-terminal sequence of harmonin is highly conserved across species (98.8% similarity between *Homo sapiens* and *Sus scrofa domesticus*), this antibody does not discriminate between endogenous porcine and human harmonin proteins. Quantification across treatment groups showed no statistically significant difference in harmonin expression at the protein level (Fig. 8), indicating that the vector-transduced cells of the WT porcine retina did not translate the abundant human *USH1C* mRNA transcripts into stable harmonin protein. This finding may indicate a regulatory mechanism that limits the expression of harmonin to the endogenous level or a defect in the OT_USH_101 vector.

**Figure 9.**
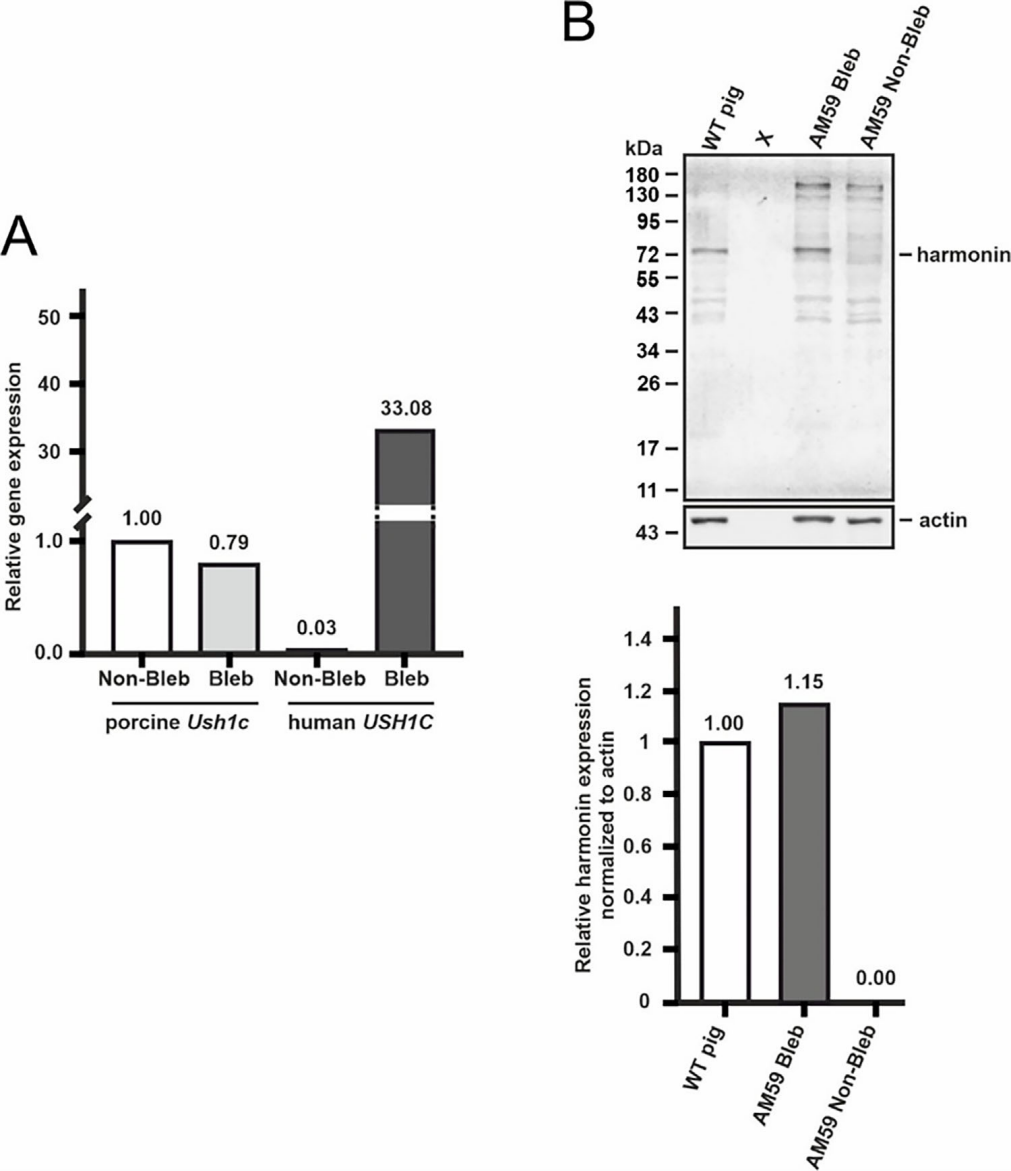
Pilot study proving USH1C/harmonin expression after subretinal injection of OT_USH_101. (**A**) qPCR comparing the endogenous Ush1c and ectopic USH1C expression in the non-bleb and bleb areas. (**B**) Anti-harmonin Western blot of protein lysates from the non-bleb and bleb areas of AM59 with WT porcine retina as a control. Lower bar plot: Quantification of anti-harmonin bands relative to the anti-actin loading control bands.

To determine whether the OT_USH_101 vector contains the correct human *USH1C* cDNA sequence necessary to express harmonin protein and the vector does was correct we performed a pilot study with one available USH1C^R31*^ pig, which does not express endogenous harmonin protein.^20^ We subretinally injected 200 µL of high-dose OT_USH_101 vector into one eye of the animal. After sacrifice, two months post-injection, qPCR analysis revealed as expected endogenous porcine *Ush1c*_a1,^20^ but also high ectopic expression of human *USH1C_a1* transcripts, confirming the data obtained in experiments with WT pigs (Fig. 9A). Western blot analysis of the protein lysates demonstrated harmonin protein expression in the bleb region of the vector-treated USH1C^R31*^ retina, whereas no harmonin protein was detected in the non-bleb region (Fig. 9B). The quantification of harmonin bands in the absence of any harmonin protein confirmed a slightly higher harmonin expression in the treated USH1C^R31*^ pig eye when compared to untreated WT control.

### Müller Glia Activation After Subretinal Vector Injections

**Figure 10.**
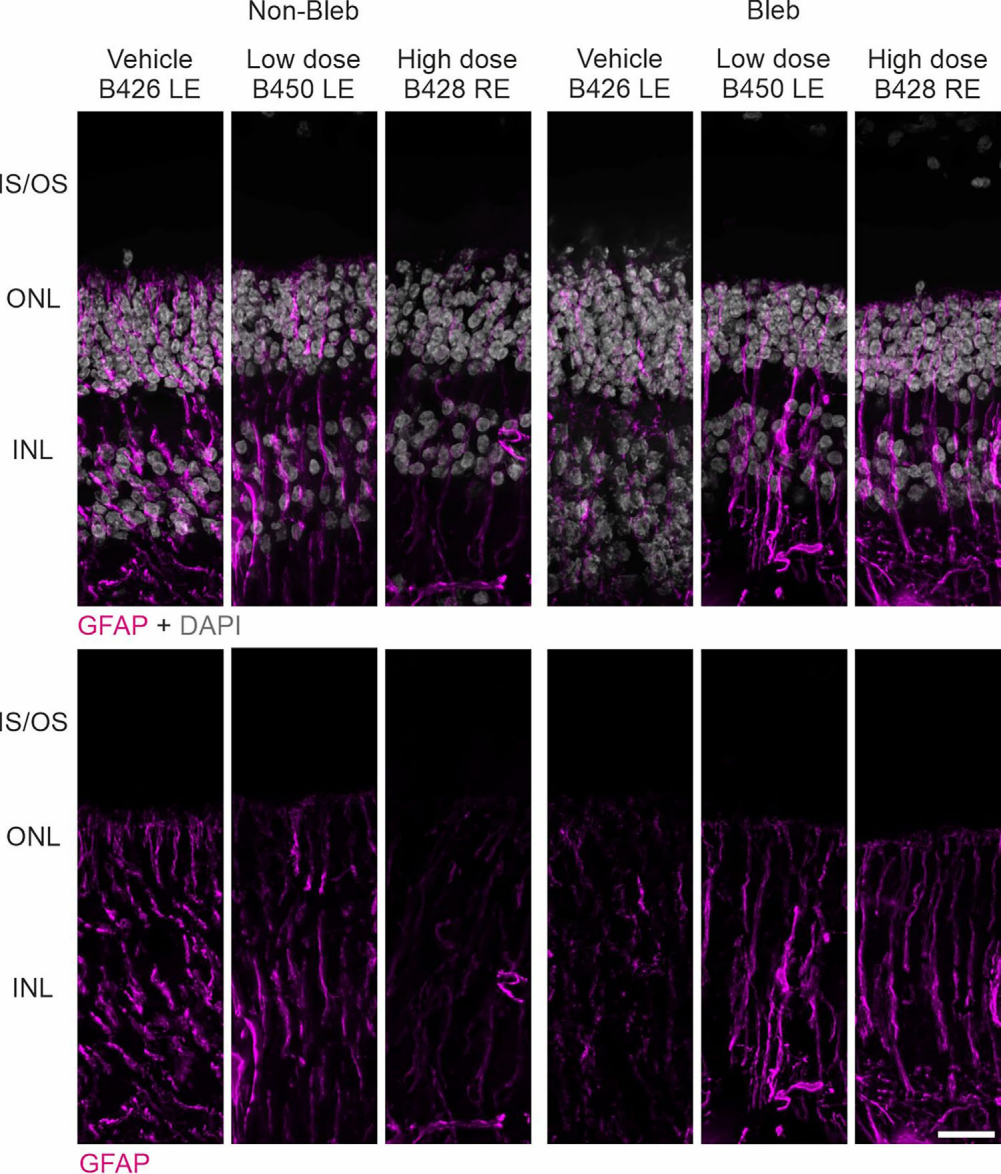
Immunohistochemical analysis of gliosis by immunostaining for GFAP (*magenta*) in WT porcine retinas after application of vehicle and vectors at low and high doses. Upper part: sections was counterstained for nuclear DNA in the outer nuclear layer (ONL) and inner nuclear layer (INL) with DAPI (*gray*). Lower part: Single GFAP channel for better visualization of the GFAP expression. IS, inner segment; OS, outer segment. *Scale bars*: 20 µm.

**Figure 11.**
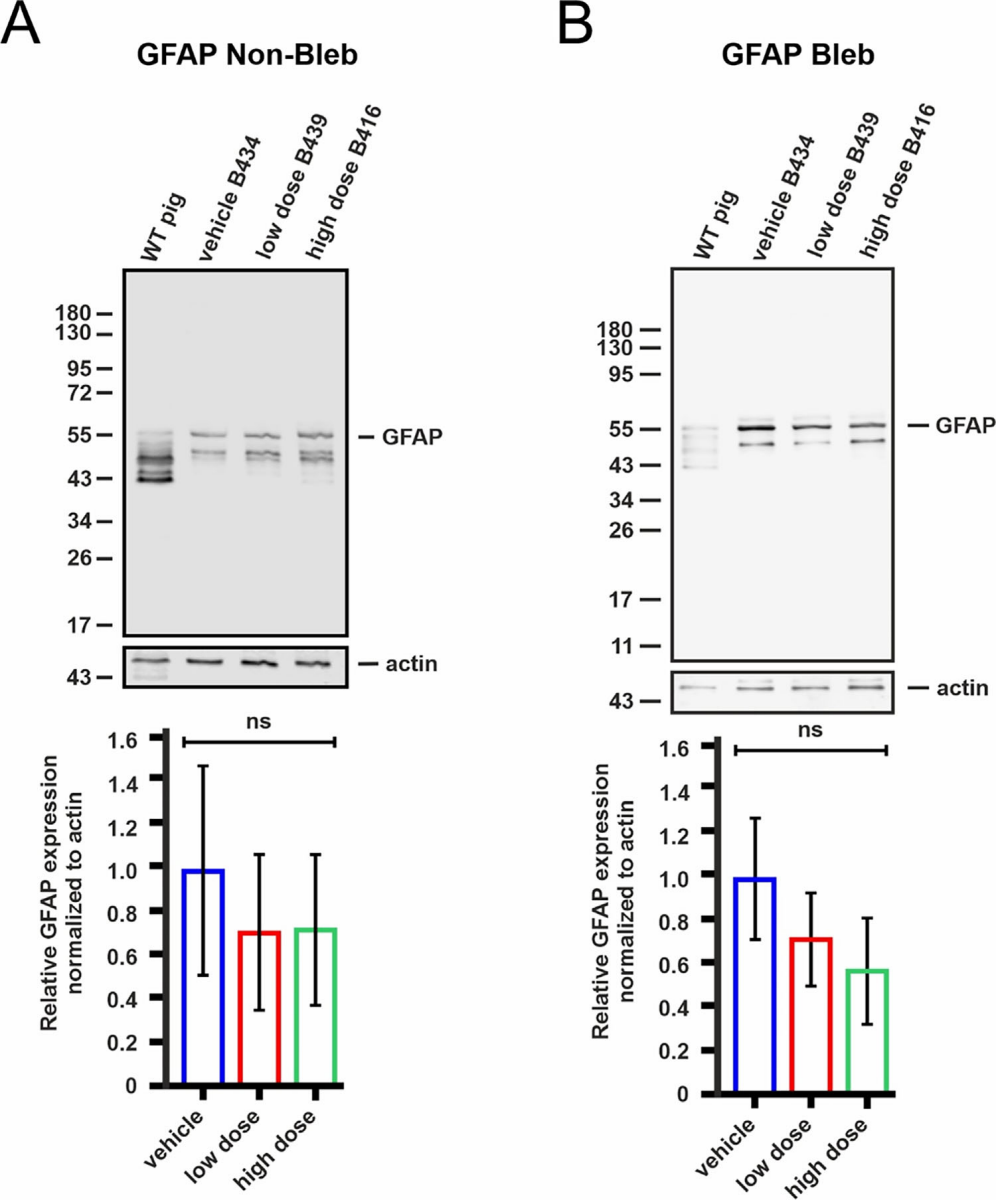
GFAP expression analysis by Western blots of porcine retinas after application of vehicle (*blue*) and vectors at low (*red*) and high (*green*) doses. (**A**) Representative anti-GFAP Western blot of protein lysates from the non-treated (non-bleb) areas of porcine retinas; *lower bar plot*: quantification of anti-GFAP bands relative to the anti-actin loading control bands from three Western blots of three porcine retinas from different animals. (**B**) Representative anti-GFAP Western blot of protein lysates from the treated (bleb) areas of porcine retinas; *lower bar plot*: quantification of anti-GFAP bands relative to the anti-actin loading control bands of three Western blots of three porcine retinas from different animals. No significant differences in GFAP protein expression were found in the non-treated (non-bleb) areas (one-way analysis of variance) or in the treated (bleb) areas (Kruskal-Wallis test). Numbers on Western blots represent molecular weight markers in kDa. ns, not significant.

We also studied whether the treatment leads to retinal gliosis. Anti-GFAP immunohistochemistry suggested some Müller glia activation in both the treated and untreated retinal areas (Fig. 10). However, the quantification of anti-GFAP Western blot bands showed the highest levels in the vehicle group (Fig. 11).

## Discussion

Early-phase data provide evidence that subretinal delivery of OT_USH_101 leads to the expression of transgenic human USH1C_a1 mRNA in the treated retina and is well tolerated in the lower dose treatment arm, whereas moderate histopathological changes were observed in the higher dose treatment arm. No statistically significant changes in TRT were seen in any of the groups over an eight-week period (vehicle, low dose, high dose) despite the surgical trauma that comes with subretinal injections, including (i) penetration of the neurosensory retina with a 41G needle, (ii) forced separation of photoreceptors from their interdigitating retinal pigment epithelium, and (iii) stretching of the neurosensory retina to accommodate the excess volume into the potential subretinal space within a matter of seconds. Although the clinical picture of a macula-off retinal detachment is not a good comparison for various reasons (etiology, mechanism), vitreoretinal surgeons generally agree that a subretinal injection would lead to a decline in function and thinning of the retina (as the photoreceptor outer segments are lost). In the ideal scenario, this loss is transient in nature as the photoreceptor outer segments renew, and no other damage is done. Studies in many different animal models have shown that iatrogenic detachment/reattachment comes with significant and complex changes to the retina not only in terms of outer segment renewal but also changes to the retinal synaptic circuitry in the inner retina and activation of glia cells throughout the retina.^25^

Direct toxic effects on the retina typically have a biphasic effect on total retinal thickness. In the acute phase, retinal toxicity results in increased retinal thickness due to an inflammatory response. Christoforidis et al.^26^ observed significant thickening and hyperreflectivity in the inner retinal layers three days post-quinine poisoning, resembling the changes seen in acute central retinal artery occlusion.^27^ In the acute phase, inflammatory retinochoroidal lesions lead to thickening and disorganization of the retinal layers.^28^^,^^29^ However, the chronic phase, occurring a few months later, is characterized by significant retinal thinning caused by cell death and atrophy of the retinal layers.^26^^–^^29^ In our study, follow-up OCT scans performed two months after gene therapy did not reveal acute or chronic retinal changes typically expected with retinal toxicity. Therefore no significant changes in TRT across all groups indicate good tolerance and absence of retinal toxicity up to the highest dose level tested.

The ff-ERG measurements can be used to monitor retinal toxicity by assessing the global functionality of photoreceptors through changes in peak times and the number of functioning photoreceptors through changes in amplitudes.^30^ Even minor toxicity can affect photoreceptors by causing a delay in their response time, measured as increased peak times.^30^ More overt toxicity can lead to photoreceptor loss, resulting in reduced amplitudes.^30^ These changes (increased peak times or reduced amplitudes) can, for example, be observed in progressive retinal degenerative diseases like retinitis pigmentosa over time.^31^^,^^32^ In our study, rod-mediated ERG changes, including delayed peak times and reduced amplitudes, were most pronounced in the vehicle group compared to both the low and high dose groups (Figs. 5A, 5B). These changes in the vehicle group are likely attributable to surgical trauma. If OT_USH_101 caused retinal toxicity, we would expect a greater increase in peak times and reduced amplitudes in the treatment groups compared to the vehicle group, which was not observed. Changes in a-wave peak time in the light-adapted 3.0 cd/m^2^ ERG were similar across all groups. Conversely, amplitudes in the light-adapted 30 Hz flicker ERG at 3 cd/m^2^ were only moderately reduced in the vehicle group, further indicating no retinal toxicity from either low or high doses of OT_USH_101. Limitations of ERG measurements in our study include modest repeatability and a relatively small sample size. However, the observed amplitudes and implicit times align with the literature.^33^^–^^35^

Histopathological changes regarding signs of retinal toxicity, inflammation and degeneration include RPE hypertrophy, loss of cell polarity, infiltration by inflammatory cells, loss of the outer nuclear layer and RPE, disrupted lamination of the outer retinal layers, and evidence of cell migration.^36^^–^^38^ In our study, there were only mild or moderate changes observed across all groups. Animals from the vehicle and low dose group showed no or minimal changes. Two out of three eyes examined from the high-dose group showed moderate retinal changes. Moderate changes were observed in only one (B416) out of three eyes in the high-dose group. It is difficult to ascertain the causality of the procedure or the vector because the changes appeared multifocal in nature and extended beyond the bleb area. But because such findings were not observed in vehicle or low-dose−treated eyes, a causal relationship with the vector cannot be ruled out. Based on the histopathological changes, the low dose of OT_USH_101 might be a safer option when treating the USH1C^R31*^ pig model in future trials. Reactive gliosis, encompassing reactive responses of Müller glia and microglial cells, was observed in porcine models after localized retinal detachment, which was achieved through the subretinal application of hyaluronate.^39^ In our study, gliosis was observed by immunohistochemistry in situ across all groups, with the highest levels in the vehicle group, likely because of retinal detachment from the subretinal vehicle application. However, we did not observe significant increases in GFAP expression by quantitative Western blots between the groups in the treated and untreated areas of the retina. If retinal toxicity had been induced by gene therapy using low or high doses, an increase in gliosis would have been expected in the treated areas of these groups; however, this was not observed in our study. Histological changes and GFAP activation were noted in the same WT pig (B416) without any other clinical signs of inflammation or toxicity.

Transgene expression of the human *USH1C_a1* coding sequence originating from the OT_USH_101 vector showed a robust mRNA expression level in the treated areas. Interestingly, the ectopic expression of human *USH1C_a1* transcripts did not impact the levels of porcine *Ush1c* expression level. Somewhat surprisingly, there was no increase in USH1C/harmonin protein expression when quantified by Western blots. The antibody used here targeted the N-terminal region of the harmonin protein and does not discriminate between human and porcine USH1C protein. Consequently, one might have expected an increased signal in the treated areas because of the unchanged levels of porcine mRNA *Ush1c* expression plus the iatrogenic human *USH1C* mRNA expression after transduction with OT_USH_101. The fact that this was not observed may indicate that the transduced cells within the porcine retina are not translating the abundant human *USH1C* mRNA transcripts into stable harmonin protein in the presence of endogenous levels of porcine harmonin protein in the WT pigs. However, in the pilot study performed here in a USH1CR31* pig (M59), we observed a moderate increase in harmonin protein expression after transduction with OT_USH_101 in the absence of endogenous protein. This indicates that the transcripts of OT_USH_101 can be translated into a correct harmonin protein. It is unclear whether this indicates a self-regulating feedback loop or additional roadblocks downstream of *USH1C_a1* transcription that need to be considered in the human retina when applying OT_USH_101 in USH1C patients in a first-in-human trial.

Of interest, there was also some level of human *USH1C* mRNA expression in the samples from untreated areas. This could indicate transduction of retinal cells by refluxed vector suspension, errors in locating the treated/untreated areas, or spread of the vector beyond the bleb. Neither of these hypotheses takes away from the important finding that subretinal delivery of OT_USH_101 leads to human *USH1C_a1* expression after gene therapy in a mammalian animal model eye, similar to that of humans. In our study, subretinal injections of a 0.05 mL volume were used, which resulted in relatively small blebs and limited spread to retinal cells. However, in USH, the retina is widely affected; therefore broader exposure to gene therapy might result in better outcomes. Thus using a larger volume with the same vg could potentially increase the spread/number of cells transduced and reduce the copy number per cell, which might be beneficial to USH patients.

Developing gene therapy for USH using previous rodent models was challenging because the models accurately mimicked human inner ear deficits but exhibited minimal or no retinal symptoms.^17^^,^^40^^,^^41^ This might be associated with significant anatomical and cellular differences in the retina between mice and humans, prompting discussions on the use of alternative model species.^20^ This led to the creation of a transgenic USH1C^R31*^ pig model, which more closely mirrors the human eye's structure and function than mice, because of its similar size, comparable rod/cone ratio, and a cone-rich region known as the visual streak and—importantly—the presence of photoreceptor calyceal processes.^20^ However, before evaluating gene therapy efficacy in the USH1C^R31*^ pig model in a long-term study, it was important to determine dose-limiting toxicity to maximize efficacy and minimize adverse effects. In our study, OT_USH_101 was well tolerated at the highest dose tested. However, we assessed OT_USH_101's safety in WT pigs with normal retinas, unlike the compromised retinas in the USH1 pig model. Therefore dose-limiting toxicity in WT pigs may not mirror that in the USH1C^R31*^ pig model. OT_USH_101 is a *USH1C* gene replacement therapy; therefore it allows for the inclusion of patients with any pathogenic variant in the *USH1C* gene. This is in contrast to ASO treatment in the mouse model, which targeted only the 216G > A variant.^18^

In conclusion, our morphological, functional, and histological findings up to two months suggest that subretinal administration of OT_USH_101 is safe at the lower dose tested, with histopathological changes observed at the higher dose. Future studies using the USH1C^R31*^pig model will need to demonstrate the effectiveness and confirm the safety of OT_USH_101.

## Supplementary Material

Supplement 1
