# Supplementary material for: Safety of Human USH1C Transgene Expression Following Subretinal Injection in Wild-Type Pigs
Source: Invest Ophthalmol Vis Sci. 2025 Jan 21;66(1):48. doi: 10.1167/iovs.66.1.48 (PMC11756606; doi:10.1167/iovs.66.1.48)
Supplement: Supplement 1 [file iovs-66-1-48_s001.pdf]

Supplement

DA 0.01 responses

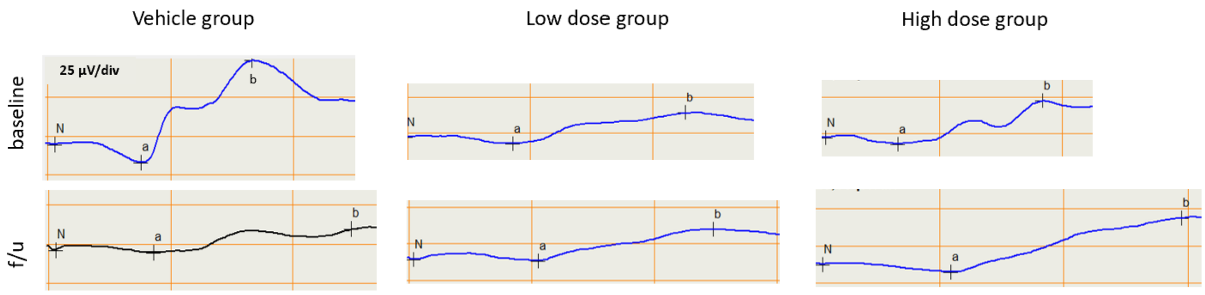

LA 3.0 responses

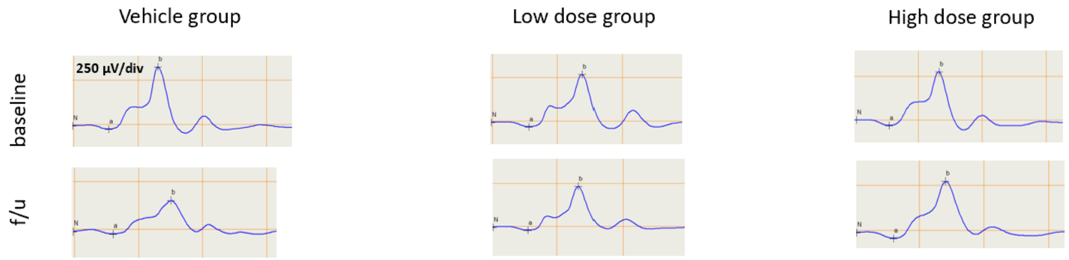

Supplementary Figure 1. Electroretinography (ERG) traces from baseline (prior to surgery) to the 2-month post-surgery follow-up (f/u). The top panel represents rod-dominated responses (dark-adapted 0.01 cd/m²), while the bottom panel shows cone-dominated responses (light-adapted 3.0 cd/m²) in the vehicle, low dose, and high dose groups.
